# CLOSED-INCISION NEGATIVE PRESSURE WOUND THERAPY (NPWT) IN OLDER PATIENTS FOLLOWING PRESSURE SORE RECONSTRUCTION

**DOI:** 10.1093/geroni/igae098.4141

**Published:** 2024-12-31

**Authors:** Young Min Jee, Seungkeun Lee, Jun Ho Park

**Affiliations:** Kangdong Sacred Heart Hospital, Seoul, Republic of Korea; Soonchunhyang University Bucheon Hospital, Bucheon, Kyonggi-do, Republic of Korea; SMG-SNU Boramae Medical Center, Seoul, Republic of Korea

## Abstract

Pressure sores significantly affect older patients, with the sacrum being especially vulnerable due to its proximity to the anus and potential for fecal contamination. Despite preventive measures, some sores still require surgical intervention. Postoperative care focuses on monitoring, hygiene, and pressure alleviation. This study assessed the efficacy of Closed-Incision Negative Pressure Wound Therapy (CI-NPWT) for postoperative wound management in patients with sacral pressure sores treated with local flaps. A retrospective analysis was performed on sacral sore patients who underwent reconstructive surgery from March 2019 through April 2023. Surgical procedures involved debridement and wound coverage using gluteal artery perforator-based fasciocutaneous flaps, followed by postoperative monitoring. Patients were grouped into either conventional monitoring or NPWT management using the INFOV.A.C. Data on patient demographics, flap metrics, fluid drainage amounts, and six-month postoperative outcomes were collected, and then analyzed with SPSS Statistics. In this study of 52 patients, 25 received NPWT and 27 received conventional dressing after surgery. While flap survival rates were 100% in both groups, the NPWT group demonstrated a significantly lower incidence of flap complication and reduced drainage amounts on the seventh postoperative day (17.2 vs. 27.8 cc, P< 0.05). The conventional dressing group exhibited one case of wound infection and three cases of dehiscence, while the NPWT group had no complications. Closed-incision NPWT may outperform conventional monitoring in treating immobilized sacral pressure sore patients following local flaps. This technique holds promise as a potential gold standard in sacral sore flap care in older patients.

